# Virtual Reality and Augmented Reality in Anatomy Education During COVID-19 Pandemic

**DOI:** 10.7759/cureus.35170

**Published:** 2023-02-19

**Authors:** Natalia Sinou, Nikoleta Sinou, Dimitrios Filippou

**Affiliations:** 1 Research and Education Institute in Biomedical Sciences, National and Kapodistrian University of Athens School of Medicine, Athens, GRC; 2 Research and Education in Biomedical Sciences, National and Kapodistrian University of Athens School of Medicine, Athens, GRC; 3 Surgery, National and Kapodistrian University of Athens School of Medicine, Athens, GRC

**Keywords:** pandemic, covid-19, anatomy education, augmented reality (ar), virtual reality (vr)

## Abstract

The pandemic of COVID-19 has radically changed the anatomy education approaches. This happens because medical students, due to the necessity of remote education, didn’t have access to cadavers, which was the principal method of dissection training. Circumstances like these encouraged the health care providers to innovate new teaching methods with the help of virtual and augmented reality to outdistance the restrictions. This review aims to examine the pioneer technological and educational tools and their usage in the future.

Detailed research was performed via the PubMed database using the following keywords “Virtual Reality (VR), Augmented Reality (AR), Anatomy Education, and COVID-19”. No further filters were used.

All the existing evidence suggests that the vast majority was negatively affected by the COVID-19 era. Using new technological methods in anatomy training could not effectively replace the absence of the traditionally used teaching methods like dissection, prosection, and lectures by physical presence.

Although the new digital anatomy teaching approaches seem to be very promising, it is not clear if they can fully replace the traditional anatomy education methods.

## Introduction and background

Historically anatomy has always been considered the foundation of medical science. Through the study of anatomy, health professionals acquire basic knowledge of the human body and its specific features. Due to the pandemic of COVID-19, the methods of anatomy training have radically changed. Since the 17^th^ century, human cadaveric dissection has been the “gold standard” of anatomy training, as it allows students to comprehend anatomical structures three-dimensionally. Further, the students were able to understand the real size and relationships of anatomical structures, making the content of lectures and books comprehensive. Even though cadaveric dissection is considered invaluable for acquiring anatomy skills, uncommon circumstances such as the COVID-19 pandemic may diminish its efficacy in anatomy training. As a result, there was a great need for discovering new educational tools for anatomy training [1].

Since technology impacts our lives a lot these days, new methods have been discovered for anatomy education. Augmented (AR) and virtual reality (VR) technologies lead to more fascinating, galvanizing, and interactive anatomy training for pre-graduates. These technologies could also contribute to anatomy education during the coronavirus era, while social distancing was required and schools did not permit students to dissect cadaveric samples [2,3].

As education turned remote, presentations and models ought to be adapted to the new online era [4-6]. Skeletons and models from bone were replaced with tutorials using advanced three-dimensional (3D) anatomy platforms and software as a visual aid. Additionally, video lectures, 3D printing materials, and ultrasound are some of the various methods technology gave to healthcare providers to study anatomy. Moreover, augmented reality (AR), in which a person is shown a computer-produced image overlaid on a portion of regular reality [7], and virtual reality (VR), in which a person experiences computer-produced surroundings/workspace entirely cut off from the rest of reality (spatial) [7] are constantly gaining more ground.

The present work aims to acquaint the several innovative educational tools for anatomy training. Further, we are going to discuss the advantages, disadvantages, and future perspectives of the aforementioned tools in the educational procedure of undergraduate students.

Materials and methods

**Figure 1:**
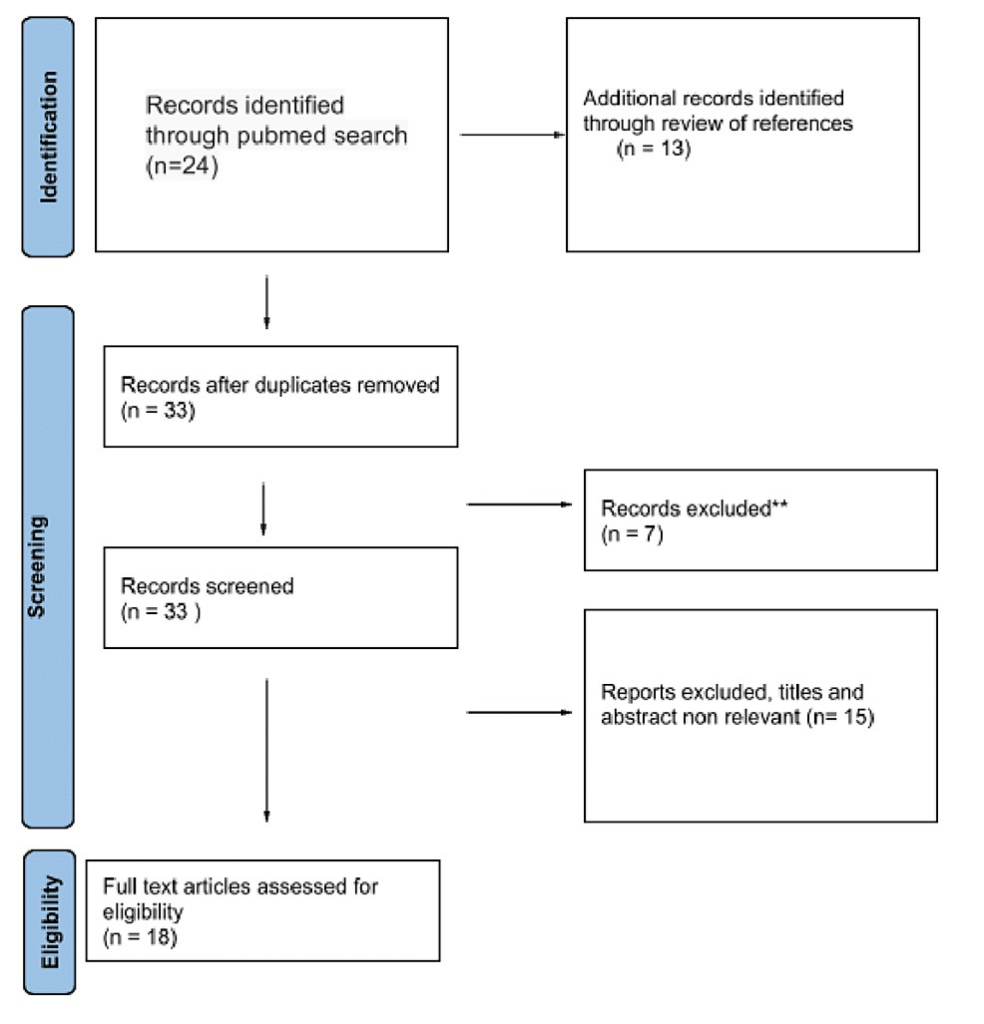
Virtual Reality and Augmented Reality in Anatomy Education During COVID-19 Pandemic PRISMA

A detailed search of the relatively published bibliography was performed in the PubMed database. The keywords used for the search were Virtual Reality (VR), Augmented Reality (AR), Anatomy, Education, COVID-19, and Pandemic. Data were mined utilizing a common data elicitation form, using the aforementioned keywords. The study was held with respect to the PRISMA-ScR guidelines. Specifically, as regards the PRISMA, the records that were identified through PubMed search were initially 24, and the additional ones through review of references were 13. So, the records after duplicates were removed, the screened ones, were 33. Also, the full-text articles assessed for eligibility were 18, and the records excluded articles, titles, and abstracts not relevant were 15. Finally, 16 references fulfilled the above-mentioned criteria and were used in the present review work (Figure 1) (Table 1).

**Table 1:** characteristics of the studies VR: Virtual reality, AR: Augmented reality

Study	Who participated	Where was the study conducted	Number of participants	Technological tools facilitated education during COVID-19	VR and AR in the future
Jiang H et al. [1]	Not mentioned	Not mentioned	114 studies	Yes	Yes
Shin M et al. [2]	AAMC associated allopathic medical schools	US	145	No	Yes
De Ponti R et al. [3]	Students attending the sixth year of the course of Medicine and Surgery	Not mentioned	122	Yes	yes
Flynn W et al. [4]	Full-time educators, 1^st^ year and 2^nd^ year medical undergraduate students, post graduate students and physician associate students	UK	Not mentioned	Yes	Yes
Moro C et al. [5]	Students of anatomy (students in biomedical and health sciences, medical students and students in other faculties)	Faculty of Health Sciences and Medicine, Bond University, Gold Coast, Queensland, Australia	59	Yes	Yes
Iwanaga J et al. [6]	Not mentioned	United States	Not mentioned	Yes	Needs further study
Iwanaga J et al. [7]	Not mentioned	Not mentioned	Not mentioned	Yes	Yes, as one of many parallel approaches
Chan V et al. [8]	Fourth-year medical students	Not mentioned	39	Yes	Needs further assessment
Alharbi Y et al. [9]	Third-year medical students of the foundation block	In the human anatomy laboratory of the College of Medicine of King Saud Bin Abdulaziz University for Health Sciences in Jeddah, Saudi Arabia	182	Not mentioned	Yes, as long as they are combined with traditional methods
Taylor L et al. [10]	Not mentioned	Not mentioned	49 papers from databases	Yes	A lot of work need to be done for these methods to be appropriate for medical students
Papalois ZA et al. [11]	Surgical science students	Olympus Simulation Lab at Guy’s Hospital	Attempted by 19 completed by 15	Yes	Yes
Iwanaga J et al. [12]	Not mentioned	Not mentioned	Not mentioned	Acceptable results	Under consideration
Singal A et al. [13]	Not mentioned	Not mentioned	Not mentioned	Not mentioned	Not mentioned
Franchi T [14]	Not mentioned	UK	Not mentioned	As a temporary measure	Yes
Nakai K et al. [15]	Second year or older medical students in Japan that have completed a traditional medical school anatomy class	In a VR workspace (Spatial) using VR headset (Oculus Quest 2, Menlo Park, CA)	30	Yes	Yes, even though there are still limitations
Zhao J et al. [16]	Not mentioned	Not mentioned	816	Not mentioned	Yes
Singal A et al. [17]	1^st^ year medical and dental students	India	80 (40 medical students and 40 dental students)	Yes	Yes
Thom ML et al. [18]	Classes of 2022, 2023 and 2024	CWRU SOM in Cleveland, Ohio, USA	552	Yes	Yes

## Review

Discussion

One of the most significant perspectives of virtual reality is the fact that users are able to interact with the virtual-generated reality. On the other hand, augmented reality is a new generation of 3D-visualized technology which provides the ability to interact with both physical and virtual objects while the latter is overlaid onto the former in real space. 

360° videos schools across the United States have begun a study about an innovative educational tool, the 360° videos [8]. The medical students that were pursuing their studies in the fourth year served as the source of the study participants, as they were at the same level of education. This method enabled students to comprehend 3D anatomy structures. However, it lags behind VR and AR in the capacity for direct interaction with objects.

Anatomage Table

A real human-size virtual dissection table offers a hands-on three-dimensional education tool [9]. This tool represents an interactive touch display measuring 2.1 m × 0.6 m and enables students to dissect a digital human cadaver virtually. Whole-body cross-sections of frozen cadavers have been pre-installed in the system. Therefore, students are able to handle anatomical structures in any slice or plane in cross-section, figuratively a selected anatomical structure, and make 3D re-enactments at multiple angles.

Gross Dissection

A similar method approved by the University of Padova, Italy, contains gross dissection on a human forearm, performed on a fresh frozen cadaver specimen.

VR Patients

Avatars representing human patients compromise an even more realistic and attractive educational tool. VR patients also provide the ability to practice soft skills such as communication and empathy while they reply in a concrete way giving the users the ability to interact with them [9,10].

HoloMentor

This method involves head-mounted display headsets that project a virtual component at the user’s eye level and at the same time, permit them to observe and interact with the environment [11]. Besides its effectiveness in remote anatomy training, it is also considered to be an arrow in the quiver of surgery training tools. This derives from the fact that HoloMentor enables surgeons to have access to the patient’s scans during an operation without looking at the monitors.

Cellphone App

A free cellphone app (Qlone) that can be used by both students and educators easily was raised during the COVID-19 pandemic when remote training was necessary. The Qlone app provides an acceptable visualization of superficial anatomical structures, but it lags in the visualization of deep structures [12].

Who Used It?

A pilot study that deals with the effectiveness of VR anatomy training during COVID-19 was accomplished in Japan with participants of second-year or older medical students who had completed a conservative anatomy class. Moreover, concerns and adaptations during COVID-19 have been analyzed by 14 anatomy departments in the UK and the Republic of Ireland (ROI) [2].

Advantages

Among various advantages of VR and AR in anatomy training, one of the most significant is that it offers the opportunity for everyone participating to approach the material remotely and the ability to shift/zoom structures of concernment at their will. The basic advantage of VR tools is that they provide multiple screens that show different images and allow comparisons between them [6]. The sense of sharing both space and time among participants is also efficient with virtual and augmented reality educational tools. Also, during COVID-19 pandemic, the major benefit of these tools is the remote accessibility with minimal equipment.

Disadvantages

One of the disadvantages of virtual reality educational tools is the fact that they have a small limit of the participants allowed to join at one time. Thus, many tables should be available or, otherwise, the lesson should be repeated multiple times. Another limitation includes the availability of associated data and files. Further, in comparison with a two-dimensional tool, VR tools do not provide the opportunity to record the meeting nor to facilitate the dialogue among the participants [6]. In addition, the constant usage of remote educational tools may affect the ability of students in communicating with patients and, generally, the human body. Another disadvantage of remote tools is the loss of concentration, neck pain, and headache or dizziness [5]. Closing, the hands-on experience that is easy in the traditional cadaveric dissection, is partially available in 3D tools and impossible in two-dimension tools (e.g., Zoom) [13].

Future Perspectives

According to the students' viewpoint [14], the demonstration of the anatomic structures through VR workspace during the lecture enhanced their understanding and contributed to the visualization of those structures in three-dimensional space [15]. Even though limitations in training via VR and AR space still exist, these tools constitute an alternative method for medical students to be educated about anatomy happening in the three-dimension space without practicing the dissection of cadavers. Although the accuracy of the 3D models is constantly being enhanced with the usage of data computed in tomography or even better with 3D scanners, the precision of the model demonstrated in the VR environment is restricted by the quantity of data that can be saved and used. For the model to be bigger, more elaborate, and more realistic, more space is needed. Thus, the use of plainer 3D models that enable undergraduate students to perceive and manage them from alternative perspectives might be more advantageous. It is hoped that the usage of VR and AR anatomy training is going to raise a wider application of these and newer technologies [16-18].

Students’ Perspective

Despite the technological progress that provides the opportunity for online distance learning, laboratory-obtained anatomical awareness, especially through human cadaver dissection, cannot be supplanted. The absence of anatomy laboratories deprived students of not only cadaver dissection but also a variety of edifying methods for learning, such as pathology specimens, bony models, and skeletons. As a result, students who practice anatomy online have no access to practical-based training materials, which makes the learning procedure less favorable and more difficult for both students and teachers. Moreover, students keep complaining that they can’t afford the cost of the necessary equipment and that the institutions should provide temporary free access during the pandemic. Aside from the difficulties stemming from students’ online anatomical education, the COVID-19 pandemic also potentially affects current anatomy students’ futures. Learning on cadaveric material is considered to be a vital part of students' evolution in their careers in the medical field, regardless of following a clinical or research path. Cadaver dissection is an irreplaceable tool for familiarizing with fine movements in a stress-free environment. For students who learn about cadavers, the donor’s body constitutes their first patient’s body, and for those who are trained in anatomy modalities, the use of virtual human resemblance represents the upcoming patient. Aside from anatomy training, students also cultivate values such as emotional intelligence, empathy, and professional behaviors through interactions and collaboration with fellow students, professors, technicians, academics, and donors. There is no doubt that anatomy training teaches students much more than just anatomy [15].

Availability of data and materials

The data were collected via the PubMed database. The information of this systematic review is available and accessible in the database mentioned using the keywords.

## Conclusions

Undeniably, it is difficult to compare the new digital educational tools to the traditional cadaveric, in-person dissection under equal and unbiased conditions. Not only does cadaveric dissection assist medical students to comprehend human-body structure and function, but the dissection experience can also further the development of teamwork, self-esteem, inter-professional and communication qualities, and ethical values. The current state of affairs in anatomy education is not the “silver bullet” for an optical subject such as anatomy. Even though students encounter numerous challenges, a shift to e-learning is nothing but necessary nowadays. As there are no indications that the digital learning era is about to end, the feedback of students may be helpful for relevant and timely modifications in electronic anatomy training. Conclusively, there is no doubt that the “gold standard” in anatomy education should be the combination of traditional and virtual or augmented reality approaches.

According to the results of the user surveys, the majority of students were partially malcontent with the virtual and augmented reality methods of anatomy training. This derives from the fact that these techniques require the latest and most expensive technological gadgets, which are not affordable for every student. Moreover, problems because of lack of strong internet connection, experience in using the new technological achievements, and financial resources turned the knowledge into an elitist privilege. So how can these difficulties be outdone so that AR and VR will be used as supplement tools in interactive anatomy training?

## References

[1] Jiang H, Vimalesvaran S, Wang JK, Lim KB, Mogali SR, Car LT (2022). Virtual reality in medical students' education: Scoping review. JMIR Med Educ.

[2] Shin M, Prasad A, Sabo G, Macnow AS, Sheth NP, Cross MB, Premkumar A (2022). Anatomy education in US Medical Schools: before, during, and beyond COVID-19. BMC Med Educ.

[3] De Ponti R, Marazzato J, Maresca AM, Rovera F, Carcano G, Ferrario MM (2020). Pre-graduation medical training including virtual reality during COVID-19 pandemic: a report on students' perception. BMC Med Educ.

[4] Flynn W, Kumar N, Donovan R, Jones M, Vickerton P (2021). Delivering online alternatives to the anatomy laboratory: Early experience during the COVID-19 pandemic. Clin Anat.

[5] Moro C, Štromberga Z, Raikos A, Stirling A (2017). The effectiveness of virtual and augmented reality in health sciences and medical anatomy. Anat Sci Educ.

[6] Iwanaga J, Kamura Y, Nishimura Y, Terada S, Kishimoto N, Tanaka T, Tubbs RS (2021). A new option for education during surgical procedures and related clinical anatomy in a virtual reality workspace. Clin Anat.

[7] Iwanaga J, Loukas M, Dumont AS, Tubbs RS (2021). A review of anatomy education during and after the COVID-19 pandemic: Revisiting traditional and modern methods to achieve future innovation. Clin Anat.

[8] Chan V, Larson ND, Moody DA, Moyer DG, Shah NL (2021). Impact of 360° vs 2D videos on engagement in anatomy education. Cureus.

[9] Alharbi Y, Al-Mansour M, Al-Saffar R, Garman A, Alraddadi A (2020). Three-dimensional virtual reality as an innovative teaching and learning tool for human anatomy courses in medical education: A mixed methods study. Cureus.

[10] Taylor L, Dyer T, Al-Azzawi M, Smith C, Nzeako O, Shah Z (2022). Extended reality anatomy undergraduate teaching: A literature review on an alternative method of learning. Ann Anat.

[11] Papalois ZA, Aydın A, Khan A (2022). HoloMentor: A novel mixed reality surgical anatomy curriculum for robot-assisted radical prostatectomy. Eur Surg Res.

[12] Iwanaga J, Terada S, Kim HJ (2021). Easy three-dimensional scanning technology for anatomy education using a free cellphone app. Clin Anat.

[13] Singal A, Bansal A, Chaudhary P (2020). Cadaverless anatomy: Darkness in the times of pandemic Covid-19. Morphologie.

[14] Franchi T (2020). The impact of the covid-19 pandemic on current anatomy education and future careers: A student's perspective. Anat Sci Educ.

[15] Nakai K, Terada S, Takahara A, Hage D, Tubbs RS, Iwanaga J (2022). Anatomy education for medical students in a virtual reality workspace: A pilot study. Clin Anat.

[16] Zhao J, Xu X, Jiang H, Ding Y (2020). The effectiveness of virtual reality-based technology on anatomy teaching: a meta-analysis of randomized controlled studies. BMC Med Educ.

[17] Singal A, Bansal A, Chaudhary P, Singh H, Patra A (2021). Anatomy education of medical and dental students during COVID-19 pandemic: a reality check. Surg Radiol Anat.

[18] Thom ML, Kimble BA, Qua K, Wish-Baratz S (2021). Is remote near-peer anatomy teaching an effective teaching strategy? Lessons learned from the transition to online learning during the Covid-19 pandemic. Anat Sci Educ.

